# Complete Genome Sequences of Three Human Oral Treponema parvum Isolates

**DOI:** 10.1128/MRA.00394-21

**Published:** 2021-07-08

**Authors:** Huihui Zeng, Rory M. Watt

**Affiliations:** a Faculty of Dentistry, The University of Hong Kong, Pok Fu Lam, Hong Kong SAR, China; Montana State University

## Abstract

Treponema parvum is a spirochete associated with human and animal oral/nonoral soft tissue infections. Here, we report the complete genome sequences of three human oral isolates of *T. parvum*, namely, ATCC 700770^T^ (OMZ 833^T^), ATCC 700773 (OMZ 842), and OMZ 843, which possess circular chromosomes of a median size of 2.63 Mb.

## ANNOUNCEMENT

Treponema parvum is a small, obligately anaerobic, strictly carbohydrate-dependent spirochete (1). Typically inhabiting human subgingival niches, it putatively plays etiological roles in periodontal disease and endodontic infections (2–4). *T. parvum* also occupies animal oral/gastrointestinal tract niches (5, 6) and has been isolated from necrotic or ulcerous tissue infections (7). Here, we report the complete genome sequences of three human clinical isolates of *T. parvum*, namely, OMZ 833^T^ (ATCC 700770^T^), OMZ 842 (ATCC 700773), and OMZ 843, obtained directly from Chris Wyss, whose group isolated and characterized these strains (1) (Table 1).

**TABLE 1:** Summary of *T. parvum* strain details, genome sequencing parameters, and major genomic features

Strain designations (1)	Clinical and geographical origin (1)	SRA accession no. for raw sequencing reads by technology		*N*_50_ (bp) for ONT sequencing reads	Depth of coverage (×)	GC content (%)	Genome size (bp)	No. of CDS^*a*^	Genome accession no.
		ONT	Illumina						
ATCC 700770^T^, OMZ 833^T^, F02FA	43-yr-old female, periodontitis lesion, Switzerland	SRR12807523	SRR12807524	10,545	921	44.0	2,658,287	2,285	CP054142
ATCC 700773, OMZ 842, 31P5C	46-yr-old female, necrotizing ulcerative gingivitis lesion, People’s Republic of China	SRR12807593	SRR12807594	10,958	1,088	44.4	2,626,237	2,287	CP054257
OMZ 843, 32COA	39-yr-old female, necrotizing ulcerative gingivitis lesion, People’s Republic of China	SRR12807630	SRR12807631	8,250	1,268	44.4	2,609,480	2,302	CP058315

a CDS, coding DNA sequences.

Axenic strains were cultured anaerobically (85% N_2_, 10% H_2_, and 5% CO_2_) at 37°C in supplemented tryptone-yeast extract-gelatin-volatile fatty acids-serum (TYGVS) medium (8). Genomic DNA was purified using QIAamp DNA mini-extraction kits (Qiagen, Germany). Long-read sequencing was performed using an Oxford Nanopore Technologies (ONT) MinION Mk1B device with an R9.4 flow cell (FLO-MIN106D). The whole-genome sequencing library was prepared using the ONT 1D genomic DNA ligation sequencing kit (SQK-LSK109) and barcoding kit (EXP-NBD104) according to the manufacturer’s protocol (v NBE_9006_v103_revP_21Dec2016). DNA was repaired using NEBNext formalin-fixed, paraffin-embedded (FFPE) DNA repair mix (New England BioLabs [NEB]) and deoxyribosyladenine (dA) tailed using the NEBNext end repair/dA-tailing module (NEB). Native barcodes were added and sequencing adapters were ligated onto the prepared ends. Libraries were washed using AMPure XP beads (Beckman Coulter). ONT reads were base called with Guppy (v3.1.5) in default mode (9), followed by demultiplexing using qcat (v1.1.0). Short-read sequencing was performed on the Illumina HiSeq X Ten (150-bp paired ends [PEs]) platform (BGI [HK] Ltd.). The short-read sequencing library was prepared by BGI (HK) Ltd. using a proprietary workflow that involved the following steps: genomic DNA was sheared (Covaris S/E210), blunt ended, and phosphorylated, single adenylate tails were added to the DNA 3′ ends, Illumina adapters were ligated, DNA was size fractionated via a magnetic bead-based approach, and selectively enriched and index tags were added by PCR. Short-read sequencing was performed on the Illumina HiSeq X Ten platform (BGI [HK] Ltd.) with an insert size of 350 bp with 150-bp paired-end reads. Illumina sequences were quality filtered using SOAPnuke (v1.5.0) (10) to remove reads containing >5% of unknown bases, >50% of bases with quality values of ≤10, and bases with read lengths of <20 bp. Adapter sequences were trimmed using Trimmomatic v0.39. Hybrid genome assembly was performed using Unicycler (v0.4.5) (11), with sequence polishing using Racon (v1.3.1) (12) and Pilon (v1.22) (13). Reads were mapped using GraphMap (v0.5.2) (14) to confirm the completeness and circularity of the assemblies. Genomes were annotated using the NCBI Prokaryotic Genomes Annotation Pipeline (PGAP) (15). Default parameters were used for all software unless otherwise specified. Sequencing, assembly, and annotation details are summarized in Table 1.

The three *T. parvum* genomes lack identifiable homologues of several key T. denticola virulence-related factors, including sialidase (TDE0471) (16) and the dentilisin protease complex (PrcB-PrcA-PrtP; TDE0760-TDE0772) (17), which is consistent with their phenotypic properties (1). Identifiable homologues of the T. denticola major surface protein (MSP; TDE0405) (18), factor H binding protein (FhbP; TDE0108) (19), and prolyl oligopeptidase (POP; TDE1195) (20) are similarly absent in *T. parvum*. The *T. parvum* type strain (ATCC 700770) genome possesses homologues of the T. denticola DNA methyltransferase (TDE0909) and restriction endonuclease (TDE0911) proteins (21), differentiating it from the ATCC 700773 and OMZ 843 strains, which appear to lack such type II restriction-modification systems.

### Data availability.

The complete *T. parvum* genome sequences and raw sequencing data were deposited in DDB/ENA/GenBank under the accession numbers CP054142 (ATCC 700770^T^), CP054257 (ATCC 700773), and CP058315 (OMZ 843) (Table 1) and under BioProject accession number PRJNA284866.
